# Supplementary material for: Homoplasy in the evolution of modern human-like joint proportions in Australopithecus afarensis
Source: eLife. 2021 May 12;10:e65897. doi: 10.7554/eLife.65897 (PMC8116054; doi:10.7554/eLife.65897)
Supplement: Supplementary file 1. — G: glenoid size; H: humeral head diameter; B: humeral biepicondylar breadth; U: ulna olecranon width; R: radial head diameter; F: femoral head diameter; Sub: femoral subtrochanteric size; A: acetabulum height; T: talar width; Sac: sacral size. Cases in which only a single upper and/or lower limb measurement was possible (DIK-1-1, BOU-VP-12/1, OH 80, KNM-ER 1503/1504, KNM-ER 3735); the reported ratio does not include a geometric mean. [file elife-65897-supp1.docx]

**Supplementary Table 1: Regional composition of the relative limb size index (RLSI) calculated for each partial skeleton.**

| **Fossil** | **Available Measurements** | | **Relative limb size index (RLSI)** |  |
| --- | --- | --- | --- | --- |
|  | **Upper Body** | **Lower Body** |  |  |
| **A.L. 288-1** | G, H, B, U, R | F, Sub, A, T, Sac | $\ln\frac{\sqrt[5]{G\times H\times B\times U\times R}}{\sqrt[5]{F\times Sub\times A\times T\times Sac}}$ |  |
| **KSD-VP-1/1** | G, B | A, Sac | $\ln\frac{\sqrt[2]{G\times B}}{\sqrt[2]{A\times Sac}}$ |  |
| **DIK-1-1** | G | T | $\ln\frac{G}{T}$ |  |
| **StW 573** | G, H, B, U, R | F, Sub, A, T | $\ln\frac{\sqrt[5]{G\times H\times B\times U\times R}}{\sqrt[4]{F\times Sub\times A\times T}}$ |  |
| **StW 431** | B, U, R | A, Sac | $\ln\frac{\sqrt[3]{B\times U\times R}}{\sqrt[2]{A\times Sac}}$ |  |
| **MH 1** | B, U | F, Sub, Sac | $\ln\frac{\sqrt[2]{B\times U}}{\sqrt[3]{F\times Sub\times Sac}}$ |  |
| **MH 2** | G, H, B, U, R | F, T, Sac | $\ln\frac{\sqrt[5]{G\times H\times B\times U\times R}}{\sqrt[3]{F\times T\times Sac}}$ |  |
| **BOU-VP 12/1** | R | Sub | $\ln\frac{R}{Sub}$ |  |
| **TM 1517** | B, U | T | $\ln\frac{\sqrt[2]{B\times U}}{T}$ |  |
| **OH 80** | R | Sub | $\ln\frac{R}{Sub}$ |  |
| **KNM-ER 1500** | U, R | Sub, T | $\ln\frac{\sqrt[2]{U\times R}}{\sqrt[2]{Sub\times T}}$ |  |
| **KNM-ER 1503/1504** | B | F, Sub | $\ln\frac{B}{\sqrt[2]{F\times Sub}}$ |  |
| **KNM-ER 3735** | B, R | Sac | $\ln\frac{\sqrt[2]{B\times R}}{Sac}$ |  |
| **KNM-WT 15000** | G, H, B, U | F, Sub, T, Sac | $\ln\frac{\sqrt[4]{G\times H\times B\times U}}{\sqrt[4]{F\times Sub\times T\times Sac}}$ |  |
| **LES 1** | H, U | F, Sub, Sac | $\ln\frac{\sqrt[2]{H\times U}}{\sqrt[3]{F\times Sub\times Sac}}$ |  |
| **LB 1** | U | F, Sub, A, T | $\ln\frac{U}{\sqrt[4]{F\times Sub\times A\times T}}$ |  |
